# Supplementary material for: Experimental evidence that female rhesus macaques (Macaca mulatta) perceive variation in male facial masculinity
Source: R Soc Open Sci. 2019 Jan 30;6(1):181415. doi: 10.1098/rsos.181415 (PMC6366174; doi:10.1098/rsos.181415)
Supplement: R Code for Analyses [file rsos181415supp1.html]

Main Analyses - Rosenfield et al. 2018


# Main Analyses - Rosenfield et al. 2018

#### *Kevin A. Rosenfield*

#### *25 August 2018*

### Install and load ggplot2

```
library(ggplot2)
```

## Read in trial data

- Data (in .csv format) is read into a dataframe, look-times are converted from frames (29/second) to seconds, and descriptive statistics for looking behavior are displayed.

```
Rhesus.Trials <- as.data.frame(read.csv("Experimental_Trial_Data.csv"))

Rhesus.Trials$Look_time <- Rhesus.Trials$Look_time / 29

Rhesus.Trials$Masc_time <- Rhesus.Trials$Masc_time / 29

Rhesus.Trials$Fem_time <- Rhesus.Trials$Fem_time / 29

summary(Rhesus.Trials[,c(5,24,30,31,32)])
```

```
##   Subject_Age     Tapped_first_R1_L0  Longer_M1_F0      Masc_time     
##  Min.   : 3.000   Min.   :0.0000     Min.   :0.0000   Min.   :0.2414  
##  1st Qu.: 5.000   1st Qu.:0.0000     1st Qu.:0.0000   1st Qu.:1.3103  
##  Median : 8.000   Median :0.0000     Median :0.0000   Median :2.2414  
##  Mean   : 8.346   Mean   :0.4486     Mean   :0.4579   Mean   :2.5669  
##  3rd Qu.:11.000   3rd Qu.:1.0000     3rd Qu.:1.0000   3rd Qu.:3.4483  
##  Max.   :22.000   Max.   :1.0000     Max.   :1.0000   Max.   :7.8276  
##     Fem_time      
##  Min.   : 0.2069  
##  1st Qu.: 1.0690  
##  Median : 2.2414  
##  Mean   : 2.5108  
##  3rd Qu.: 3.4310  
##  Max.   :10.1379
```

### Did subjects look significantly longer at one stimulus type than the other?

- Boxplots depicting look-times for masculine and feminine stimuli

```
Rhesus_Trials2 <- Rhesus.Trials[,c(31,32)]

library(reshape) 
Rhesus_Trials2 <- melt(Rhesus_Trials2)
```

```
## Using  as id variables
```

```
Rhesus_Trials2$Stim <- Rhesus_Trials2[,1]
Rhesus_Trials2$Time <- Rhesus_Trials2[,2]
Rhesus_Trials2 <- Rhesus_Trials2[,c(3,4)]

ggplot(Rhesus_Trials2, aes(x=Stim, y=Time, fill=Stim)) + ylab("Look-time (seconds)") + xlab("Image type")+ geom_boxplot() + coord_cartesian(ylim=c(0,11.2)) + scale_y_continuous(breaks=c(2, 4, 6, 8, 10)) + geom_jitter(shape=16, position=position_jitter(.2)) + theme(panel.grid.major = element_blank(), panel.grid.minor = element_blank()) + theme(legend.position = "none") + theme(axis.text=element_text(size=12), axis.title=element_text(size=14,face="bold")) + geom_text(aes(1.5, 10.5, label="NS"), size=5)
```

- The following analysis includes the entire dataset (107 trials). Wilcoxon signed-rank test (paired, non-parametric):

```
Prediction1.1 <- wilcox.test(Rhesus.Trials$Masc_time, Rhesus.Trials$Fem_time, paired = TRUE)

Prediction1.1
```

```
## 
##  Wilcoxon signed rank test with continuity correction
## 
## data:  Rhesus.Trials$Masc_time and Rhesus.Trials$Fem_time
## V = 3031, p-value = 0.4279
## alternative hypothesis: true location shift is not equal to 0
```

- We find no significant difference in masculine and feminine stimulus look-times.

### Did more subjects look longer at one stimulus type than the other?

- The following analysis includes the entire dataset (107 trials). Binomial test: is 64/105 different from chance?; 2 ties were automatically excluded:

```
Prediction1.2 <- binom.test(64, 105, p = 0.5,
           alternative = c("two.sided"),
           conf.level = 0.95);

Prediction1.2
```

```
## 
##  Exact binomial test
## 
## data:  64 and 105
## number of successes = 64, number of trials = 105, p-value = 0.0313
## alternative hypothesis: true probability of success is not equal to 0.5
## 95 percent confidence interval:
##  0.5094443 0.7032587
## sample estimates:
## probability of success 
##              0.6095238
```

- Our subjects looked longer at masculine stimuli than feminine stimuli in a significantly higher proportion of trials than would be predicted by chance.

### Was the proportion of time subjects spent looking at masculine images associated with the relative difference in masculinity between the two stimuli?

- Scatterplot depicting the relationship between masculinity difference scores and masculinity look-time percentages. Shaded area is th 95% confidence interval of the line.

```
ggplot(Rhesus.Trials, aes(x=Masc_perc, y=Masc_diff, fill=Masc_perc)) + ylab("Masculinity difference score") + xlab("Masculine image look-time\n(as proportion of total look time)") + geom_smooth(method = 'lm') + geom_point(data=NULL, cex=1.5) + theme(panel.border = element_blank(), axis.line = element_line(colour = "black")) + theme(panel.grid.major = element_blank(), panel.grid.minor = element_blank()) + theme(legend.position="none") + xlim(0,100) + theme(axis.text=element_text(size=12), axis.title=element_text(size=14,face="bold"))
```

1. The following analysis includes the entire dataset (107 trials). Linear model: predictor variable: Masculinity difference; dependent variable: masculine stilmulus look percentage:

```
Prediction2.1a <- lm(Masc_perc ~ Masc_diff, data = Rhesus.Trials); 

summary(Prediction2.1a)
```

```
## 
## Call:
## lm(formula = Masc_perc ~ Masc_diff, data = Rhesus.Trials)
## 
## Residuals:
##     Min      1Q  Median      3Q     Max 
## -40.765 -11.439   2.173   9.889  45.792 
## 
## Coefficients:
##             Estimate Std. Error t value Pr(>|t|)    
## (Intercept)  42.6019     4.5870   9.287 2.43e-15 ***
## Masc_diff     0.2903     0.1317   2.204   0.0297 *  
## ---
## Signif. codes:  0 '***' 0.001 '**' 0.01 '*' 0.05 '.' 0.1 ' ' 1
## 
## Residual standard error: 16.46 on 105 degrees of freedom
## Multiple R-squared:  0.04421,    Adjusted R-squared:  0.0351 
## F-statistic: 4.856 on 1 and 105 DF,  p-value: 0.02973
```

- The within-pair Masculinity difference explains a significant proportion of the variability in the masculine stilmulus look percentage.

2. Now we repeat the model, adding raw masculinity score as an additional predictor variable:

```
Prediction2.1b <- lm(Masc_perc ~ Masc_diff + Masc_score, data = Rhesus.Trials); 

summary(Prediction2.1b)
```

```
## 
## Call:
## lm(formula = Masc_perc ~ Masc_diff + Masc_score, data = Rhesus.Trials)
## 
## Residuals:
##     Min      1Q  Median      3Q     Max 
## -41.580 -10.587   0.753  10.104  44.906 
## 
## Coefficients:
##             Estimate Std. Error t value Pr(>|t|)  
## (Intercept)  78.5787    37.8634   2.075   0.0404 *
## Masc_diff     0.3785     0.1608   2.354   0.0205 *
## Masc_score  -17.8964    18.6960  -0.957   0.3407  
## ---
## Signif. codes:  0 '***' 0.001 '**' 0.01 '*' 0.05 '.' 0.1 ' ' 1
## 
## Residual standard error: 16.47 on 104 degrees of freedom
## Multiple R-squared:  0.05255,    Adjusted R-squared:  0.03433 
## F-statistic: 2.884 on 2 and 104 DF,  p-value: 0.06037
```

- The within-pair Masculinity difference explains a significant proportion of the variability in the masculine stilmulus look percentage when controlling for the raw score of the masculinie image.
- The masculine image’s score, however, does not explain a significant proportion of the variability in the masculine stilmulus look percentage when relative scores are controlled.

### Did subjects look significantly longer at one stimulus type than the other in a subset of trials with high relative masculinity differences?

1. First, the dataset is subsetted into two data frames, based on a binary variable indicating whether the masculinity difference is high (n=54) or low (n=53).

- Boxplots depicting look-times for masculine and feminine stimuli in the high and low masculinity differences groups (requires changing data from horizontally to vertically oriented)

```
Rhesus_Trials2 <- Rhesus.Trials[,c(21, 31,32)]

Rhesus_Trials2_high <- subset(Rhesus_Trials2, MD_cat == 1)
Rhesus_Trials2_low <- subset(Rhesus_Trials2, MD_cat == 0)
Rhesus_Trials2_high <- Rhesus_Trials2_high[,c(2,3)]
Rhesus_Trials2_low <- Rhesus_Trials2_low[,c(2,3)] 

Rhesus_Trials2_high <- melt(Rhesus_Trials2_high)
```

```
## Using  as id variables
```

```
Rhesus_Trials2_low <- melt(Rhesus_Trials2_low)
```

```
## Using  as id variables
```

```
Rhesus_Trials2_high$Stim <- Rhesus_Trials2_high[,1]
Rhesus_Trials2_high$Time <- Rhesus_Trials2_high[,2]
Rhesus_Trials2_high <- Rhesus_Trials2_high[,c(3,4)]

Rhesus_Trials2_low$Stim <- Rhesus_Trials2_low[,1]
Rhesus_Trials2_low$Time <- Rhesus_Trials2_low[,2]
Rhesus_Trials2_low <- Rhesus_Trials2_low[,c(3,4)]


ggplot(Rhesus_Trials2_high, aes(x=Stim, y=Time, fill=Stim)) + ylab("Look-time (seconds)") + xlab("Image type")+ geom_boxplot() + coord_cartesian(ylim=c(0,11.2)) + scale_y_continuous(breaks=c(2, 4, 6, 8, 10)) + geom_jitter(shape=16, position=position_jitter(.2)) + theme(panel.grid.major = element_blank(), panel.grid.minor = element_blank()) + theme(legend.position = "none") + theme(axis.text=element_text(size=12), axis.title=element_text(size=14,face="bold")) + geom_text(aes(1.5, 10.5, label="*"), size=10)
```

```
ggplot(Rhesus_Trials2_low, aes(x=Stim, y=Time, fill=Stim)) + ylab("Look-time (seconds)") + xlab("Image type") + geom_boxplot() + coord_cartesian(ylim=c(0,11.2)) + scale_y_continuous(breaks=c(2, 4, 6, 8, 10)) + geom_jitter(shape=16, position=position_jitter(.2)) + theme(panel.grid.major = element_blank(), panel.grid.minor = element_blank()) + theme(legend.position = "none") + theme(axis.text=element_text(size=12), axis.title=element_text(size=14,face="bold")) + geom_text(aes(1.5, 10.5, label="NS"), size=5)
```

- Subsetting of data for analyses

```
High_diff <- subset(Rhesus.Trials, MD_cat == 1)
Low_diff <- subset(Rhesus.Trials, MD_cat == 0)
```

2. Summaries for the two resulting datasets:

- High differences

```
summary(High_diff[,c(5,24,30,31,32)])
```

```
##   Subject_Age     Tapped_first_R1_L0  Longer_M1_F0   Masc_time     
##  Min.   : 3.000   Min.   :0.000      Min.   :0.0   Min.   :0.6207  
##  1st Qu.: 5.000   1st Qu.:0.000      1st Qu.:0.0   1st Qu.:1.3276  
##  Median : 8.000   Median :0.000      Median :0.5   Median :2.2586  
##  Mean   : 8.444   Mean   :0.463      Mean   :0.5   Mean   :2.8135  
##  3rd Qu.:11.000   3rd Qu.:1.000      3rd Qu.:1.0   3rd Qu.:3.7845  
##  Max.   :22.000   Max.   :1.000      Max.   :1.0   Max.   :7.8276  
##     Fem_time     
##  Min.   :0.2414  
##  1st Qu.:0.9828  
##  Median :1.5862  
##  Mean   :2.3193  
##  3rd Qu.:3.2931  
##  Max.   :8.0345
```

- Low differences

```
summary(Low_diff[,c(5,24,30,31,32)])
```

```
##   Subject_Age     Tapped_first_R1_L0  Longer_M1_F0      Masc_time     
##  Min.   : 3.000   Min.   :0.000      Min.   :0.0000   Min.   :0.2414  
##  1st Qu.: 5.000   1st Qu.:0.000      1st Qu.:0.0000   1st Qu.:1.3103  
##  Median : 8.000   Median :0.000      Median :0.0000   Median :2.2414  
##  Mean   : 8.245   Mean   :0.434      Mean   :0.4151   Mean   :2.3155  
##  3rd Qu.:11.000   3rd Qu.:1.000      3rd Qu.:1.0000   3rd Qu.:3.1724  
##  Max.   :18.000   Max.   :1.000      Max.   :1.0000   Max.   :5.3793  
##     Fem_time      
##  Min.   : 0.2069  
##  1st Qu.: 1.3448  
##  Median : 2.3793  
##  Mean   : 2.7059  
##  3rd Qu.: 3.9310  
##  Max.   :10.1379
```

3. Now, Wilcoxon signed-rank tests (paired, non-parametric) are repeated, once for the low masculinity differences group, once for the high masculinity differences group:

- High differences

```
Prediction2.2a <- wilcox.test(High_diff$Masc_time, High_diff$Fem_time, paired = TRUE)
```

- We find that subjects looked significantly longer at masculine than feminine stimuli in the subset of trials with high masculinity differences.
- Low differences

```
Prediction2.2b <- wilcox.test(Low_diff$Masc_time, Low_diff$Fem_time, paired = TRUE)
```

- We find no significant difference in masculine and feminine stimulus look-times in the subset of trials with low masculinity differences.

### Check for confounding variables

- Does group co-membership between subjects and stimulus males influence looking behavior?


```
No_Groupmate <- subset(Rhesus.Trials, Groupmates=="None", select = "Masc_perc")

Fem_Groupmate <- subset(Rhesus.Trials, Groupmates=="Fem", select = "Masc_perc")

Masc_Groupmate <- subset(Rhesus.Trials, Groupmates=="Masc", select = "Masc_perc")

Both_Groupmate <- subset(Rhesus.Trials, Groupmates=="Both", select = "Masc_perc")

Groupmates <- merge(No_Groupmate, Fem_Groupmate)
```

2. Analyses (Wilcoxon signed-rank) testing whether subjects’ masculine stimulus look-time percentages differed when the subject and 1) the feminine stimulus male was a groupmate, 2) the masculine stimulus male was a groupmate, or 3) both stimulus males were groupmates; each is compared to trials in which neither was a groupmate:

```
confound_masc_groupmate <- wilcox.test(Masc_Groupmate$Masc_perc, No_Groupmate$Masc_perc, paired = FALSE); confound_masc_groupmate
```

```
## 
##  Wilcoxon rank sum test with continuity correction
## 
## data:  Masc_Groupmate$Masc_perc and No_Groupmate$Masc_perc
## W = 543.5, p-value = 0.9025
## alternative hypothesis: true location shift is not equal to 0
```

```
counfound_fem_groupmate <- wilcox.test(Fem_Groupmate$Masc_perc, No_Groupmate$Masc_perc, paired = FALSE); counfound_fem_groupmate
```

```
## 
##  Wilcoxon rank sum test with continuity correction
## 
## data:  Fem_Groupmate$Masc_perc and No_Groupmate$Masc_perc
## W = 378, p-value = 0.9839
## alternative hypothesis: true location shift is not equal to 0
```

```
confound_both_groupmxate <- wilcox.test(Both_Groupmate$Masc_perc, No_Groupmate$Masc_perc, paired = FALSE); confound_both_groupmxate
```

```
## 
##  Wilcoxon rank sum test with continuity correction
## 
## data:  Both_Groupmate$Masc_perc and No_Groupmate$Masc_perc
## W = 345, p-value = 0.1983
## alternative hypothesis: true location shift is not equal to 0
```

- We find no evidence that co-goup membership affected subjects’ looking behavior.

## Read in facial masculinity, color, and luminance data

```
Rhesus.Masculinity <- as.data.frame(read.csv("Facial_Masculinity_Data.csv"))

Rhesus.Masculinity$Color <- as.numeric(as.character(Rhesus.Masculinity$Color))
```

```
## Warning: NAs introduced by coercion
```

```
Rhesus.Masculinity$Luminance <- as.numeric(as.character(Rhesus.Masculinity$Luminance))
```

```
## Warning: NAs introduced by coercion
```

- Subset data into three datasets: One including all stumli, one including masculine stimuli only, and one including feminine stimuli only

```
Stimuli <- subset(Rhesus.Masculinity, M2_F1_Not0 > 0, select = c("Age","Color","Luminance","Masculinity_score"))

Masc_Stimuli <- subset(Rhesus.Masculinity, M2_F1_Not0 == 2, select = c("Age","Color","Luminance","Masculinity_score"))

Fem_Stimuli <- subset(Rhesus.Masculinity, M2_F1_Not0 == 1, select = c("Age","Color","Luminance","Masculinity_score"))
```

1. Are facial masculinity scores related to age?

```
Age_corr <- cor.test(Stimuli$Masculinity_score, Stimuli$Age, method = "spearman");
```

```
## Warning in cor.test.default(Stimuli$Masculinity_score, Stimuli$Age, method
## = "spearman"): Cannot compute exact p-value with ties
```

```
Age_corr
```

```
## 
##  Spearman's rank correlation rho
## 
## data:  Stimuli$Masculinity_score and Stimuli$Age
## S = 1230.7, p-value = 0.7545
## alternative hypothesis: true rho is not equal to 0
## sample estimates:
##        rho 
## 0.07464449
```

```
Age_means <- wilcox.test(Masc_Stimuli$Age, Fem_Stimuli$Age, paired = FALSE);
```

```
## Warning in wilcox.test.default(Masc_Stimuli$Age, Fem_Stimuli$Age, paired =
## FALSE): cannot compute exact p-value with ties
```

```
Age_means
```

```
## 
##  Wilcoxon rank sum test with continuity correction
## 
## data:  Masc_Stimuli$Age and Fem_Stimuli$Age
## W = 60.5, p-value = 0.4438
## alternative hypothesis: true location shift is not equal to 0
```

- Among stimuli, age is not signicantly correlated with facial masculinity, and age does not differ significantly between masculine and feminine stimuli.

2. Are facial masculinity scores related to facial color?

```
Color_corr <- cor.test(Stimuli$Masculinity_score, Stimuli$Color, method = "spearman");

Color_corr
```

```
## 
##  Spearman's rank correlation rho
## 
## data:  Stimuli$Masculinity_score and Stimuli$Color
## S = 1424, p-value = 0.7673
## alternative hypothesis: true rho is not equal to 0
## sample estimates:
##         rho 
## -0.07067669
```

```
Color_means <- wilcox.test(Masc_Stimuli$Color, Fem_Stimuli$Color, paired = FALSE);

Color_means
```

```
## 
##  Wilcoxon rank sum test
## 
## data:  Masc_Stimuli$Color and Fem_Stimuli$Color
## W = 44, p-value = 0.6842
## alternative hypothesis: true location shift is not equal to 0
```

- Among stimuli, facial color is not signicantly correlated with facial masculinity, and facial color does not differ significantly between masculine and feminine stimuli.

3. Are facial masculinity scores related to facial luminance?

```
Luminance_corr <- cor.test(Stimuli$Masculinity_score, Stimuli$Luminance, method = "spearman");
```

```
## Warning in cor.test.default(Stimuli$Masculinity_score, Stimuli$Luminance, :
## Cannot compute exact p-value with ties
```

```
Luminance_corr
```

```
## 
##  Spearman's rank correlation rho
## 
## data:  Stimuli$Masculinity_score and Stimuli$Luminance
## S = 884.83, p-value = 0.1492
## alternative hypothesis: true rho is not equal to 0
## sample estimates:
##       rho 
## 0.3347123
```

```
Luminance_means <- wilcox.test(Masc_Stimuli$Luminance, Fem_Stimuli$Luminance, paired = FALSE);
```

```
## Warning in wilcox.test.default(Masc_Stimuli$Luminance,
## Fem_Stimuli$Luminance, : cannot compute exact p-value with ties
```

```
Luminance_means
```

```
## 
##  Wilcoxon rank sum test with continuity correction
## 
## data:  Masc_Stimuli$Luminance and Fem_Stimuli$Luminance
## W = 71.5, p-value = 0.1123
## alternative hypothesis: true location shift is not equal to 0
```

\*Among stimuli, facial luminance is not signicantly correlated with facial masculinity, and facial luminance does not differ significantly between masculine and feminine stimuli.

### Analyses of possible confounding variables reveals no evidence that facial masculinity is confounded by the age, facial color, or facial luminance of stimulus males.

#### Because we seperated the odd number of trials into high (N=54) and low (N=53) masculinity differences groups arbitrarily, to test robustness of our results, we repeated these analyses with trials seperated into alternative high (N=53) and low (N=54) groups (one trial moved the high to the low group).

```
High_diff_extra <- subset(Rhesus.Trials, MD_cat2 > 0)
Low_diff_extra <- subset(Rhesus.Trials, MD_cat2 < 1)
```

#### Summaries for the two resulting datasets:

```
summary(High_diff_extra[,c(5,24,30,31,32)])
```

```
##   Subject_Age   Tapped_first_R1_L0  Longer_M1_F0      Masc_time     
##  Min.   : 3.0   Min.   :0.0000     Min.   :0.0000   Min.   :0.6207  
##  1st Qu.: 5.0   1st Qu.:0.0000     1st Qu.:0.0000   1st Qu.:1.3448  
##  Median : 8.0   Median :0.0000     Median :1.0000   Median :2.2759  
##  Mean   : 8.4   Mean   :0.4727     Mean   :0.5091   Mean   :2.8088  
##  3rd Qu.:11.0   3rd Qu.:1.0000     3rd Qu.:1.0000   3rd Qu.:3.7414  
##  Max.   :22.0   Max.   :1.0000     Max.   :1.0000   Max.   :7.8276  
##     Fem_time     
##  Min.   :0.2414  
##  1st Qu.:1.0000  
##  Median :1.4828  
##  Mean   :2.2997  
##  3rd Qu.:3.2759  
##  Max.   :8.0345
```

```
summary(Low_diff_extra[,c(5,24,30,31,32)])
```

```
##   Subject_Age     Tapped_first_R1_L0  Longer_M1_F0      Masc_time     
##  Min.   : 3.000   Min.   :0.0000     Min.   :0.0000   Min.   :0.2414  
##  1st Qu.: 5.000   1st Qu.:0.0000     1st Qu.:0.0000   1st Qu.:1.2931  
##  Median : 8.000   Median :0.0000     Median :0.0000   Median :2.2414  
##  Mean   : 8.288   Mean   :0.4231     Mean   :0.4038   Mean   :2.3110  
##  3rd Qu.:11.000   3rd Qu.:1.0000     3rd Qu.:1.0000   3rd Qu.:3.1810  
##  Max.   :18.000   Max.   :1.0000     Max.   :1.0000   Max.   :5.3793  
##     Fem_time      
##  Min.   : 0.2069  
##  1st Qu.: 1.3707  
##  Median : 2.3966  
##  Mean   : 2.7341  
##  3rd Qu.: 3.9483  
##  Max.   :10.1379
```

#### Now, Wilcoxon signed-rank tests (paired, non-parametric) are repeated, once for the low masculinity differences group, once for the high masculinity differences group:

```
Prediction2.2a_extra <- wilcox.test(High_diff$Masc_time, High_diff$Fem_time, paired = TRUE);

Prediction2.2a_extra
```

```
## 
##  Wilcoxon signed rank test with continuity correction
## 
## data:  High_diff$Masc_time and High_diff$Fem_time
## V = 1024, p-value = 0.01554
## alternative hypothesis: true location shift is not equal to 0
```

- We still find that subjects looked significantly longer at masculine than feminine stimuli in the subset of trials with high masculinity differences.

```
Prediction2.2b_extra <- wilcox.test(Low_diff$Masc_time, Low_diff$Fem_time, paired = TRUE);

Prediction2.2b_extra
```

```
## 
##  Wilcoxon signed rank test with continuity correction
## 
## data:  Low_diff$Masc_time and Low_diff$Fem_time
## V = 526, p-value = 0.2007
## alternative hypothesis: true location shift is not equal to 0
```

- We still find no significant difference in masculine and feminine stimulus look-times in the subset of trials with low masculinity differences.
